# Study on automatic detection of wheat spike grain number based on deep learning

**DOI:** 10.3389/fpls.2026.1724501

**Published:** 2026-02-25

**Authors:** Hecang Zang, Yanjing Wang, Shengwei Wang, Shuai Ren, Yandong Yang, Jie Zhang, Qing Zhao

**Affiliations:** 1 Institute of Agricultural Information Technology, Henan Academy of Agricultural Sciences, Zhengzhou, China; 2 Huanghuaihai Key Laboratory of Intelligent Agricultural Technology, Ministry of Agriculture and Rural Areas, Zhengzhou, China; 3 School of Life Science, Zhengzhou Normal University, Zhengzhou, China; 4 Agricultural Information Institute, Chinese Academy of Agricultural Sciences, Beijing, China; 5 Xinxiang Academy of Agricultural Sciences, Xinxiang, China

**Keywords:** deep learning, field phenotype, spike grains, target detection, wheat

## Abstract

In wheat breeding, the number of spike grains is a key indicator for evaluating wheat yield, and timely and accurate detection of wheat spike grain is of great practical significance for yield estimation. However, in actual field production, the counting of spike grain still relies on manual counting after threshing, which poses problems such as complex measurement processes, time-consuming and laborious. At present, achieving automated and intelligent detection of wheat spike grain still faces significant challenge. Therefore, the focus of this study is to use the most advanced computer vision technology for fast and automatic detection of wheat spike grain. During the wheat filling stage, a total of 936 wheat spike grain images were collected, and these images were expanded through data augmentation to ultimately obtain 3700 wheat spike grain images. According to the partition ratio of the small scale dataset, 80% of the 3700 images are used for training, 10% for validation, and the remaining 10% for testing. This study selected six state-of-the-art deep learning models: YOLOv8n, YOLOv8s, YOLOv8m, YOLOv8l, YOLOv8x, and Faster R-CNN. In all wheat spike grain test, YOLOv8n showed high precision, recall, mAP50, and mAP50-95, with values of 96.8%, 96.8%, 98.9%, and 58.4%, respectively. The precision of other models was 96.7% for YOLOv8m, 96.5% for YOLOv8s, 96.3% for YOLOv8l, 96.2% for YOLOv8x, and 95.7% for Faster R-CNN. YOLOv8n not only has a lower number of parameters, FLOPs, inference time, model size, and GPU memory usage, as well as higher detection precision in wheat spike grain counting tasks, fully meet the spike grain counting requirements of wheat breeding. The multi-scale feature fusion and lightweight computing of YOLOv8n help improve model performance, and its performance is better compared to other deep learning models. This study designed and implemented a WeChat mini program for wheat spike grain counting, so as to achieve automatic detection and counting of wheat spike grains, which provided valuable reference for grain detection, counting, and yield estimation of other crops.

## Introduction

Wheat is the most widely planted food crop in the world, providing a staple food for one-third of the world’s population and having significant implications for global food production and security (Tester and Langridge, 2010; Chauhan et al., 2020; Zhao et al., 2021; Wen et al., 2022; Zang et al., 2025). According to official statistics from the Food and Agriculture Organization of the United Nations (FAO), the global wheat planting area in 2022 was 221 million hectares, with a total output of 780 million tons (FAO, 2023). Wheat is an important grain crop in China, it is the world’s largest producer of wheat and bears the responsibility of ensuring global food security (Zang et al., 2022). However, food security is facing severe global challenges. According to the FAO, in order to meet the survival needs of approximately 9.7 billion people worldwide by 2050, the total global food production needs to increase by more than 69% on the existing basis. Therefore, timely and accurate prediction of wheat yield is the key to ensuring food supply security and sustainable agricultural development.

The important factors that contribute to wheat yield include the number of spikes per unit area, the number of grains per spike, and the thousand grain weight. Among them, the number of grains per spike is a key factor in wheat yield formation and a core parameter for yield estimation (Koua et al., 2021). In actual production, the wheat spike grain counting method mainly involves threshing the wheat grains and then manually counting the number of grains, which limits the monitoring frequency, scale, and accuracy of wheat spike grain (Tester and Langridge, 2010). However, this method is inefficient, labor-intensive, and time-consuming. Improving the automatic detection and counting methods of wheat spike grain, which has become a major scientific problem that urgently needs to be solved in the field of wheat phenotype omics. In view of this, the development of fast and accurate counting method for wheat spike grains in field environments, which is of great practical significance for wheat breeding screening and field yield estimation.

In recent years, with the deep integration of computer vision and machine learning technology, scholars at home and abroad have made breakthrough progress in the research of wheat grain number phenotype detection. Al-Tam et al. (2013) developed the P-TRAP software, which uses traditional image processing algorithms such as local adaptive threshold segmentation, and two-dimensional skeleton analysis to estimate the number of rice spike grain. However, before measuring spike grain traits, threshing is required, and it is not suitable for varieties with denser spike grain. Gong et al. (2018) used wavelet frequency feature analysis based on rice panicle contour to estimate the number of grains in rice panicles. However, the high computational complexity of their algorithm limits the applicability of high-throughput phenotype acquisition scenarios. Wu et al. (2019) developed a rice grain counting method, and their constructed linear regression model achieved grain counting accuracy of over 96% and 97% for japonica and indica rice, respectively, achieving estimation of rice grain number per panicle. Olgun et al. (2016) constructed an automated wheat grain classification system based on support vector machine, and K-means clustering algorithm, with an accuracy rate of 88.33%. Alharbi et al. (2018) used vegetation extraction color index to segment wheat spike images, combined with Gabor filter feature enhancement, K-means clustering segmentation, and regression modeling methods, to achieve wheat spike detection with an average accuracy of 90.7%. Fernandez-Gallego et al. (2019) counted wheat spikes using thermal imaging and RGB images, and the counting results of the two methods were highly consistent, with an R^2^ of 0.83. Tan et al. (2020) used a simple linear iterative clustering algorithm to perform superpixel segmentation on wheat images in the field, extracting morphological contours and color space features of wheat spikes, and achieving an accuracy of 94.01% in estimating the number of wheat spikes. The above research preprocesses wheat spike images through image processing techniques to obtain features such as color, contour, and texture. Then, a suitable model is constructed based on the wheat spike features to achieve wheat spike recognition, segmentation, and counting. However, this method still has shortcomings, and there is relatively little research on the number of wheat spike grain, a complete process for wheat grain counting and yield estimation has not yet been formed.

With the rapid development of artificial intelligence, breakthrough progress has been made in wheat spike image detection technology based on deep learning (Madec et al., 2019; He et al., 2020), achieving excellent performance in detection accuracy and inference speed (Zhou et al., 2018; Khoroshevsky et al., 2021; Lu et al., 2022; Wang et al., 2022). Numerous studies have shown that the combination of deep learning and image processing techniques has excellent performance, and has been widely applied in tasks such as wheat spike recognition, segmentation, and counting. Object detection algorithms mainly include single-stage object detection algorithms and two-stage object detection algorithms, from the R-CNN series of two-stage object detection to the SSD and YOLO series of single-stage object detection algorithms (Redmon and Farhadi, 2017; Redmon et al., 2016; Redmon and Farhadi, 2018; Bochkovskiy et al., 2020; Ultralytics, 2022). Compared to two-stage object detection algorithms, single-stage object detection algorithms use regression mechanisms to achieve end-to-end prediction, significantly improving computational efficiency while maintaining high accuracy. Zhao et al. (2021) developed a wheat spike detection method based on improved YOLOv5, with an average accuracy of 94.1%, significantly improving the problem of false detection and missed detection of wheat spike images in occluded scenes. Wu et al. (2023) improved the YOLOv7 model and DeepSort algorithm, proposing a wheat spike counting method. The mAP of the improved YOLOv7 was 96.2%, which was 2.5 percentage points higher than the original model. Meng et al. (2023) developed the YOLOv-MA model, which improved the feature extraction ability of wheat spike targets by introducing small-scale detection layers and convolutional block attention modules, achieving an mAP of 93.86%. Wu et al. (2020) built a Faster R-CNN model based on the TensorFlow framework and optimized the wheat grain detection and counting model using transfer learning methods, with an average accuracy of 0.91. Xu et al. (2023) constructed a CBAM-HRNet model incorporating CBAM for wheat grain segmentation and counting, with an accuracy of 92.04%. The above research indicates that algorithms based on deep learning significantly improve the accuracy of wheat spike grain detection. However, there are still limitations in terms of detection speed and practical application, and it is urgent to overcome the problem of accurate counting of wheat spike grain.

In response to the core challenges of small grain size, high density, and severe occlusion in wheat grain counting, YOLOv8 enhances its ability to detect local features of dense small-sized grains through multi-scale feature fusion. Its anchor free mechanism simplifies the prediction process and reduces the target miss rate in dense areas. In contrast, Faster R-CNN adopts a two-stage detection framework, which cascades the first round of coarse selection and the second round of fine tuning to more accurately separate and locate grain targets in overlapping areas, thereby alleviating the problem of feature confusion. Compared to traditional machine learning methods, deep learning models represented by YOLOv8 have fundamental advantages. Traditional machine learning methods use phased rule-based concatenation, which is prone to failure when faced with complex variations such as grain adhesion and uneven lighting. YOLOv8 achieves unified optimization of feature learning, target localization, and classification through end-to-end deep convolutional networks, and can automatically learn high-level semantic features. For grain adhesion, the model can capture subtle differences in boundary texture; For lighting changes, its multi-layer nonlinear transformation has the ability to represent lighting invariance, thereby achieving significant improvements in feature expression ability and environmental adaptability.

At present, deep learning technology have been widely applied in wheat grain counting research. However, related research mainly focuses on traditional grain counting after threshing wheat spike, and there are relatively few exploratory research on wheat spike grain counting. To effectively solve the problem of wheat spike grain detection and counting in complex scenarios, this study evaluates the performance of six state-of-the-art deep learning models in wheat spike grain detection, in order to select the best deep learning model, including YOLOv8n, YOLOv8s, YOLOv8m, YOLOv8l, YOLOv8x, and Faster R-CNN.

## Materials and methods

### Research area

**Figure 1:**
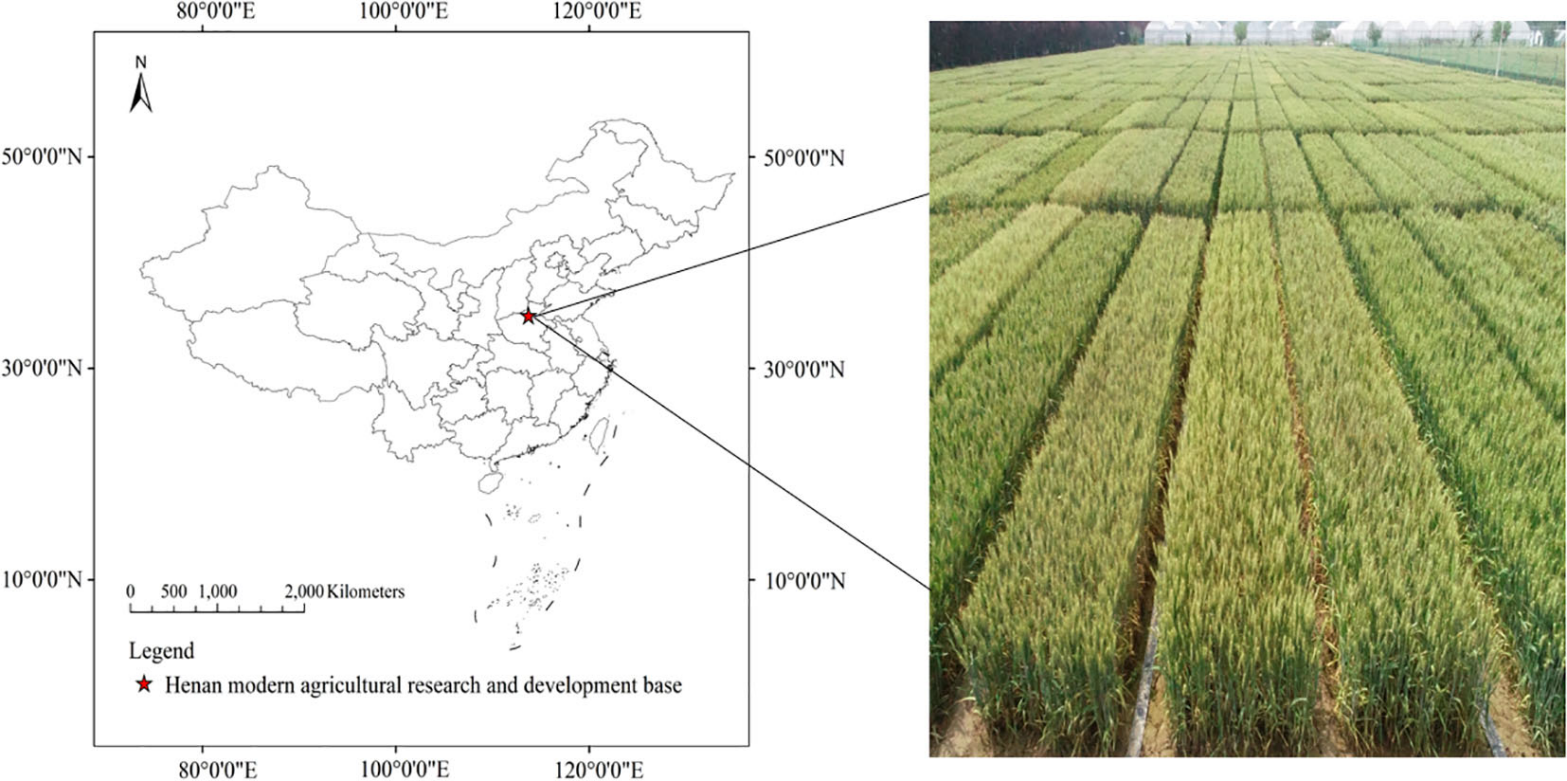
Research area.

The wheat regional experiment is located in Henan Modern Agriculture Research and Development Base, Yuanyang County, Xinxiang City, Henan Province, China, at longitude 113°41´E and latitude 35°0´N, as shown in **Figure 1**. Yuanyang County is under the jurisdiction of Xinxiang City, Henan Province. It belongs to a semi-arid and semi humid warm temperate continental monsoon climate, with a flat terrain and convenient drainage and irrigation. The tested soil is sandy loamy tidal soil, and the previous crop in the experimental field is summer corn. The average annual temperature is 14.5°C, and the average annual rainfall is 660 mm, mainly concentrated from June to September. The experimental materials are all varieties participating in the national Huanghuai southern region trial, a total of 80 wheat varieties, sown on October 18, 2023, with a sowing rate of 195 kg/hm^2^. The experiment adopts a randomized block design with 3 replicates, 6 rows per plot, a row spacing of 20 cm, and a plot area of 12 m^2^. During the wheat season, 750 kg/hm^2^ of base fertilizer mixed fertilizer and 7500 kg/hm^2^ of decomposed cow manure are applied. At the standing stage, 150 kg/hm^2^ of urea is applied, and irrigation is carried out at once, the nitrogen fertilizer ratio of base fertilizer to top fertilizer is 6:4. Other management technique is consistent with conventional field production.

### Data collection

**Figure 2:**
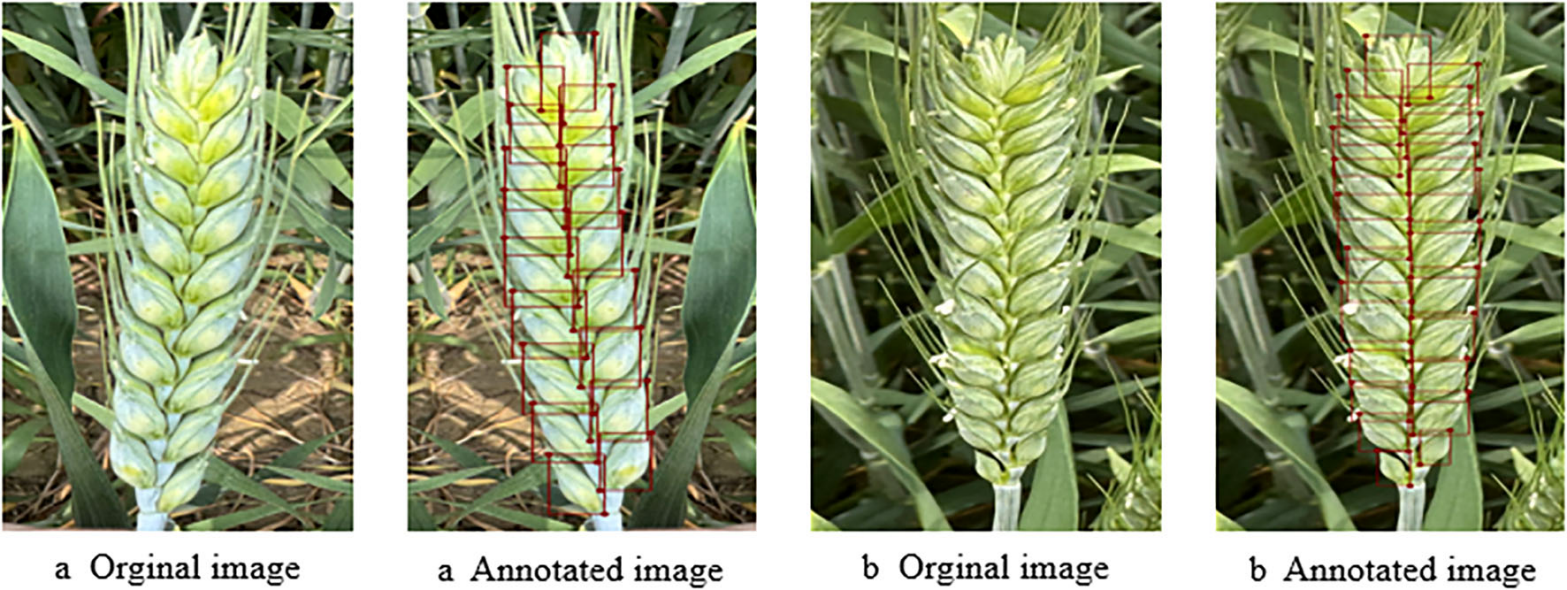
Example of wheat spike grain image.

The experiment begins during the wheat filling stage, and wheat spike images are collected according to the requirement. During the grain filling period, the color of wheat spikes gradually changes from green to yellow, and the grains become increasingly plump. Especially in the middle and late stages of grain filling, there is a significant difference in color between the wheat spike and the stem and leaves, which facilitates clear differentiation of the grains on the wheat spike (Misra et al., 2021). The data obtained in this stage helps optimize the preprocessing process of wheat spike images and provides technical support for building and validating deep learning models. However, in the process of wheat spike image acquisition, factors such as lighting, equipment parameters, and focusing accuracy may lead to exposure abnormalities or shadow interference, affecting the quality of spike and grain imaging, thereby increasing the complexity of later spike and grain image processing and potentially reducing model accuracy (Alirezazadeh et al., 2023). Select clear weather to collect images of wheat spikes, with specific shooting times from am 9:00-11:00 and pm 3:00-6:00, the image format is JPG. The image acquisition device is a smartphone, using the iPhone 15 Pro Max model to capture wheat spike images. The main camera is 48 million pixels, with an aperture of f/1.78. The phone’s shooting mode is set to optical zoom in AI mode, with a resolution of 2796×1290 pixels and an image format of JPG. When collecting wheat grain images in the field, keep the smartphone parallel to the wheat grain and adjust the distance to make the wheat grain completely centered on the preview screen, making it easier to obtain clear and complete grain images. After screening and removing unqualified and redundant images, 936 wheat grain images were finally obtained. An example of wheat grain image is shown in **Figure 2**.

### Dataset construction

**Figure 3:**
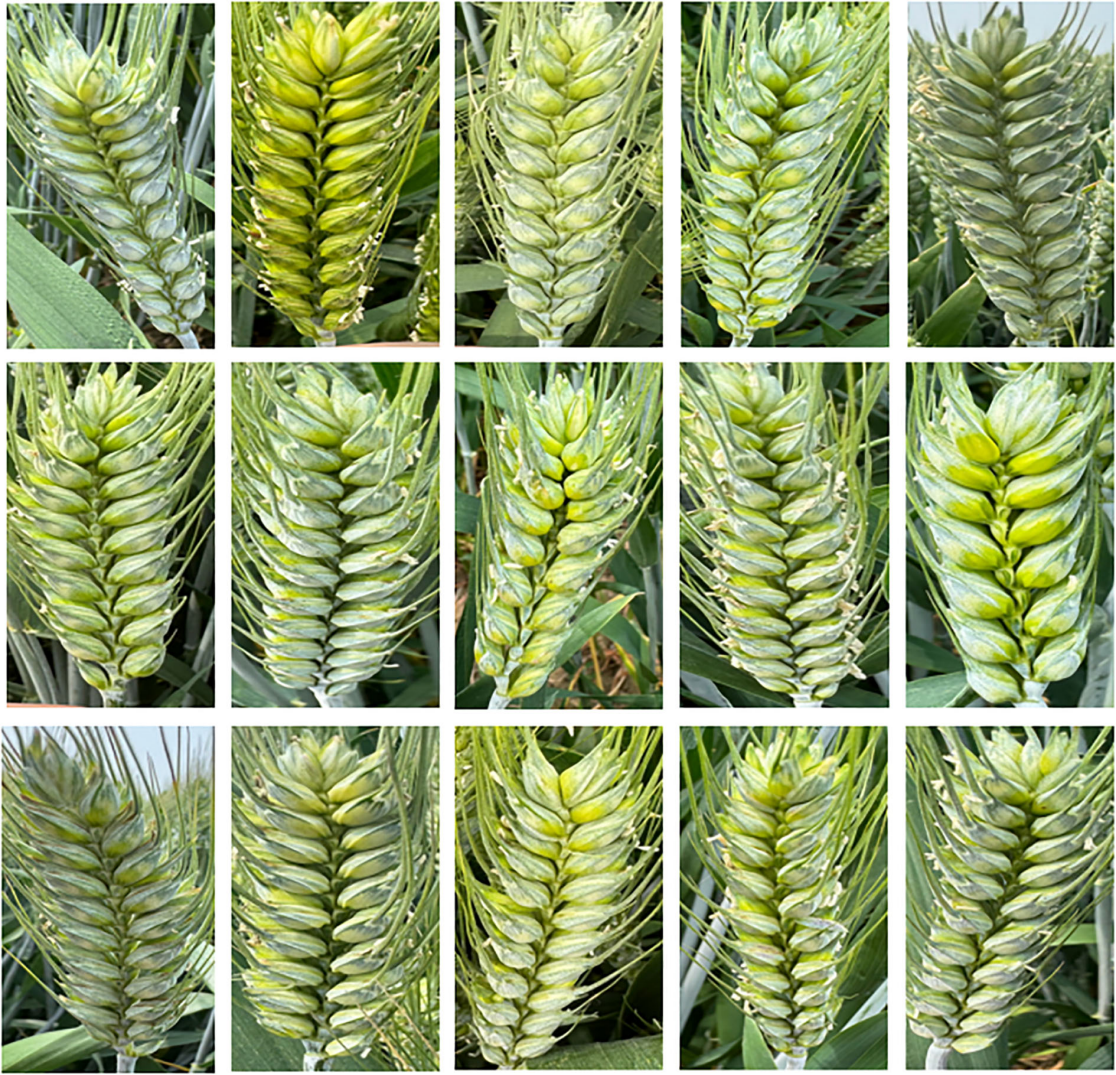
Original and annotated image of wheat spike grain.

This study constructed a wheat spike grain image dataset based on captured spike grain images. Firstly, use the labeling software Labelme to annotate the contour of wheat grain hulls with bounding boxes. Some images and their annotation examples are shown in **Figure 3**. Subsequently, 936 original wheat grain images were divided into training set, testing set, and validation set in an 8:1:1 ratio. Image flipping, rotation, brightness balance, and Gaussian noise were used for data augmentation, resulting in 3700 wheat grain images. The training set contained 2960 images, while the testing and validation sets contained 370 images. In order to improve data processing efficiency and facilitate deep learning model training, the original images are uniformly cropped to a resolution of 640×640 pixels.

### Deep learning models

**Figure 4:**
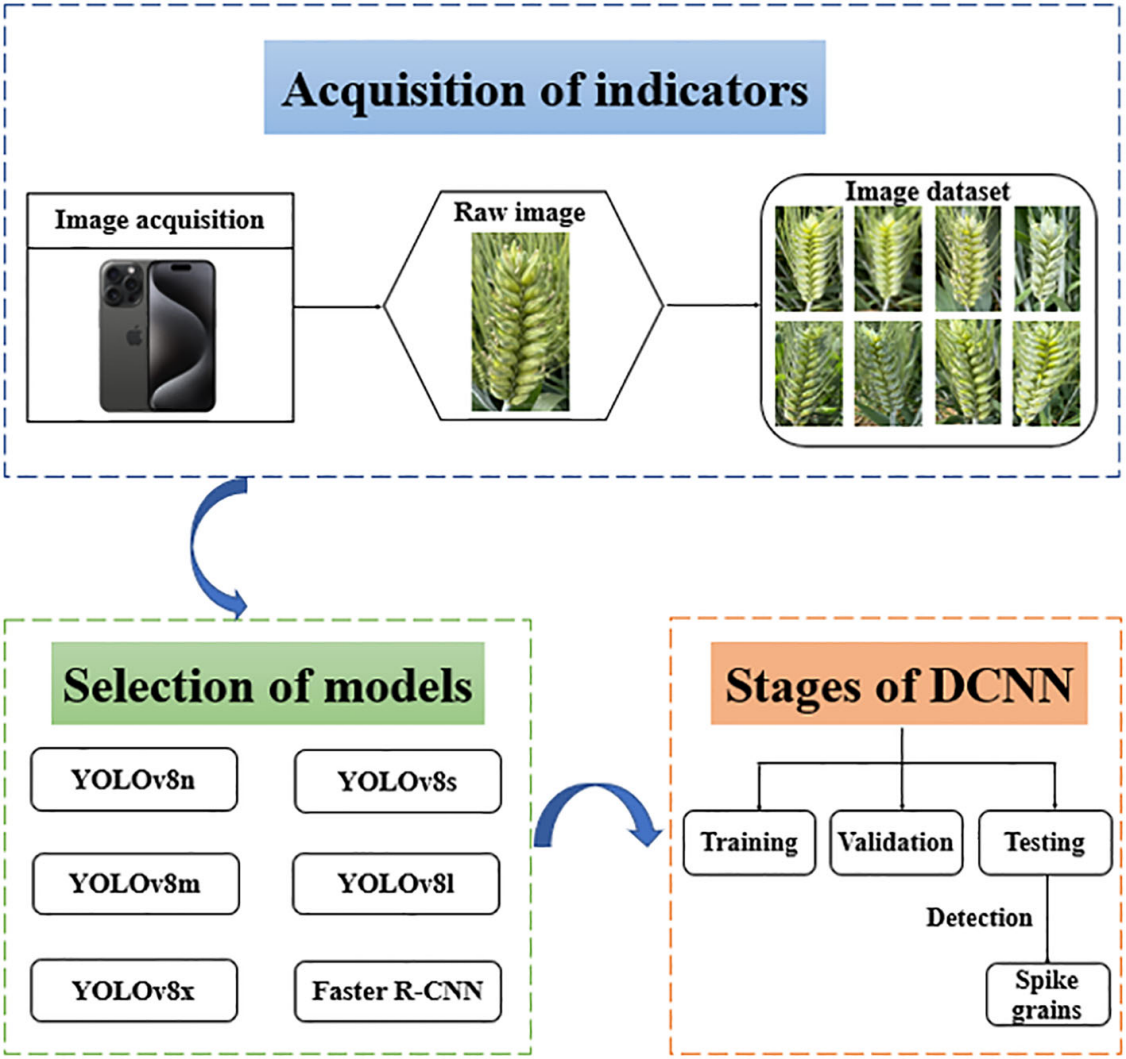
Framework for detecting wheat spike grain using deep learning models.

Deep learning models consist of convolutional layers, pooling layers, and fully connected layers, which perform feature extraction and classification on input data (Modi et al., 2023). The convolutional layer serves as the fundamental component of the model, extracting local features of the input image through convolution operations. The pooling layer is usually placed after the convolutional layer to reduce the spatial dimension of the feature map, improve the computational efficiency and feature robustness of the model. The fully connected layer is located at the end of the network, integrating the abstract features extracted by the convolutional layer and pooling layer to perform the final classification or regression task. Especially in crop phenotype research, convolutional neural networks (CNN) have shown outstanding performance in tasks such as image classification, object detection, and segmentation due to their powerful feature extraction capability, and have become the core technology for analyzing complex field agricultural images. This study selected a total of six state-of-the-art deep learning models, including YOLOv8n, YOLOv8s, YOLOv8m, YOLOv8l, YOLOv8x, and Faster R-CNN. The architecture of the deep learning models used for wheat spike grain detection is shown in **Table 1**. All models were pretrained using transfer learning, and regardless of changes in shooting altitude and weather conditions, as long as they were trained and parameter optimized appropriately in a controllable operating environment, the models could maintain good accuracy and effectiveness. The overall process of model selection, parameter tuning, training, validation, and testing is shown in **Figure 4**.

**Table 1:** Architecture of deep learning models selection for wheat spike grain detection.

Models	Backbone	Image input size	No of layers	ReLU layer	Max pooling layer	Convolution layer	Fully connected layer
YOLOv8n	CSPDarknet	640×640×3	225	–	3	64	–
YOLOv8s	CSPDarknet	640×640×3	225	–	3	84	–
YOLOv8m	CSPDarknet	640×640×3	295	–	3	103	–
YOLOv8l	CSPDarknet	640×640×3	365	–	3	121	–
YOLOv8x	CSPDarknet	640×640×3	365	–	3	141	–
Faster R-CNN	ResNet50	640×640×3	214	56	1	62	3

YOLOv8 effectively integrates shallow high-resolution detail features with deep strong semantic features through a multi-scale feature fusion mechanism, significantly improving the localization and recognition ability of small-sized grains. Meanwhile, the hierarchical feature extraction structure of YOLOv8 gradually expands the receptive field, enhancing its ability to capture multidimensional features of small-sized grains. As the smallest model in the YOLOv8 series, YOLOv8n has extremely fast inference speed and low computing power requirements, making it suitable for deployment on mobile devices or embedded systems, and can meet the practical application needs of rapid detection and counting of wheat grains in the field.

Object detection is the core task of computer vision, which aims to identify objects in images or videos and locate their spatial positions. In recent years, the mainstream algorithm YOLOv8 has become a hot topic in the research and application of this field. CNN automatically learn to extract features from low order textures, edges to high-order shapes and semantics from raw inputs through multiple layers of trainable convolution kernels. This not only significantly reduces the dependence on artificial feature engineering, but also enhances the model’s ability to handle large-scale, heterogeneous, and high-dimensional data (Kim et al., 2022). In addition, the end-to-end training mechanism of deep learning models enables them to have strong cross domain transfer learning capabilities, which can effectively cope with various complex scenarios such as dynamic lighting, partial occlusion, and multimodal data fusion. Deep learning models achieve automatic extraction and representation learning of high-order abstract features by constructing multi-layer nonlinear network structures. Compared to traditional machine learning methods that rely on manually designed features, deep learning has demonstrated significant advantages in the field of computer vision due to its powerful end-to-end learning capability.

### Model detection and manual statistical evaluation

This study conducted manual statistics and measurements based on images and actual measurements of spike grains. The number of spike grains based on images was manually counted for the grains that could be clearly identified in each wheat spike image; based on actual measurements, the number of spike grains in a single wheat plant is manually counted. In terms of algorithm evaluation, the study used linear regression analysis to analyze the correlation between the number of spike grains detected by the model and the manually calculated number of spike grains.

### Experimental configuration

The hardware configuration of this experiment is equipped with Intel (R) Core i7–10600 CPU, with a main frequency of 2.90 GHz; NVIDIA GeForce RTX3090 GPU, workstation with video memory capacity of 24GB. The software framework uses PyTorch to input the dataset in batches and iterate through all batches to complete a single iteration. The optimizer selects Adam, the training batch size is set to 8, the total number of iterations is 1000, the initial learning rate is 0.001, and it gradually decays to 0.0001 as the number of iterations increases.

### Evaluation indicators

Evaluation indicators are quantitative standards for measuring the performance of key parameters in model tasks, providing objective basis for algorithm performance evaluation. This study aims to verify the performance of wheat grain detection and counting tasks using a multidimensional evaluation index system. This study used precision, recall, mean accuracy (mAP), coefficient of determination (R^2^), root mean square error (RMSE), parameter count, computational complexity (FLOPs), inference time, model size, and GPU memory usage as evaluation indicators for the wheat grain counting model. Precision represents the proportion of predicted spike grain to all predicted spike grain, Recall represents the proportion of predicted spike grain number to actual spike grain number, mAP represents the area under the precision and recall curves, averaged over the intersection to union threshold. In this study, the model evaluation was conducted using the following settings: IoU threshold of 0.7, confidence threshold of 0.25, and average protocol of single class average. MAP50–95 represents the average precision mean of the IoU threshold within the range of 0.5-0.95 (with a step size of 0.05), strictly following the standard conventions of the COCO dataset. R^2^ can reflect the correlation between the model’s statistical wheat spike grain number and the measured wheat spike grain number, and is used to measure the quality of the model. RMSE can demonstrate the robustness of the model in detecting wheat grain counts. The calculation of precision, recall, mAP, R2 and RMSE is as follows with Equations (1–5):

(1) $$Precision=\frac{TP}{TP+FP}100\%$$

(2) $$Recall=\frac{TP}{TP+FN}100\%$$

(3) $$mAP=\int_{0}^{1}P(R)dR$$

(4) $$R^{2}=1-\frac{\sum_{i}{\left(p_{i}-q_{i}\right)}^{2}}{\sum_{i}{\left(\bar{p}-q_{i}\right)}^{2}}$$

(5) $$RMSE=\sqrt{\frac{1}{N}\sum_{i=1}^{N}{\left(p_{i}-q_{i}\right)}^{2}}$$

In the formula, TP represents true positive, FP represents false positive, FN represents false negative, and TN represents true negative. n is the total number of images, and $p_{i}$ and $q_{i}$ represent the manually counted wheat spike grain number and the model detected wheat spike grain number in the i image, respectively. $\bar{p}$ represents the average number of wheat spike grains in each image.

## Results and analysis

### Accuracy, recall, and training loss of different deep learning models

**Figure 5:**
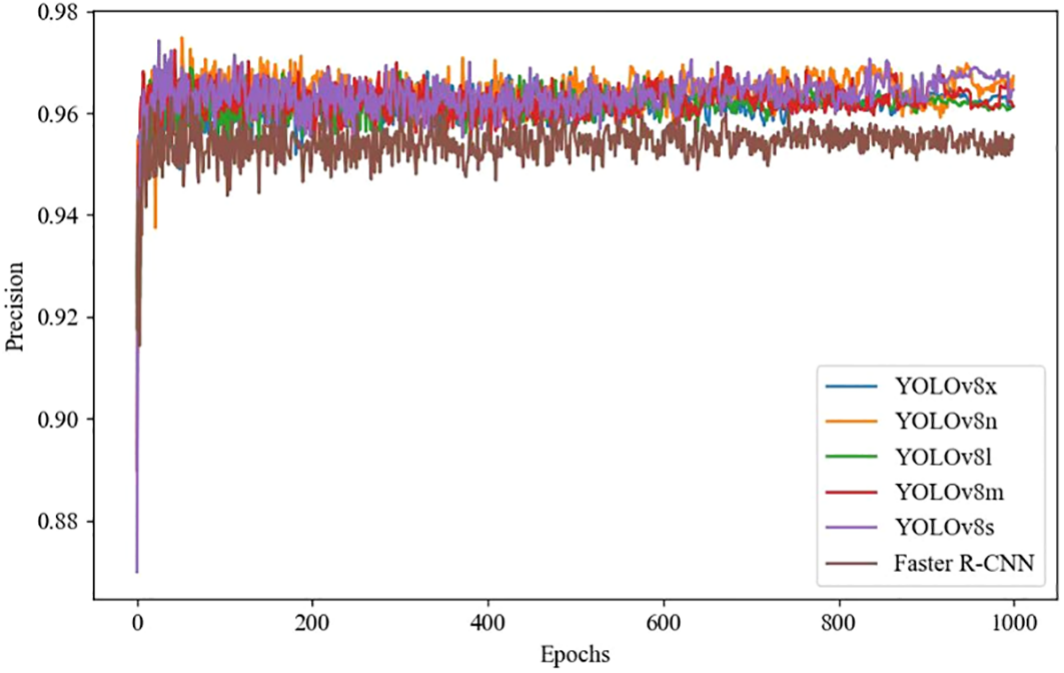
Comparison of training precision using deep learning models.

**Figure 6:**
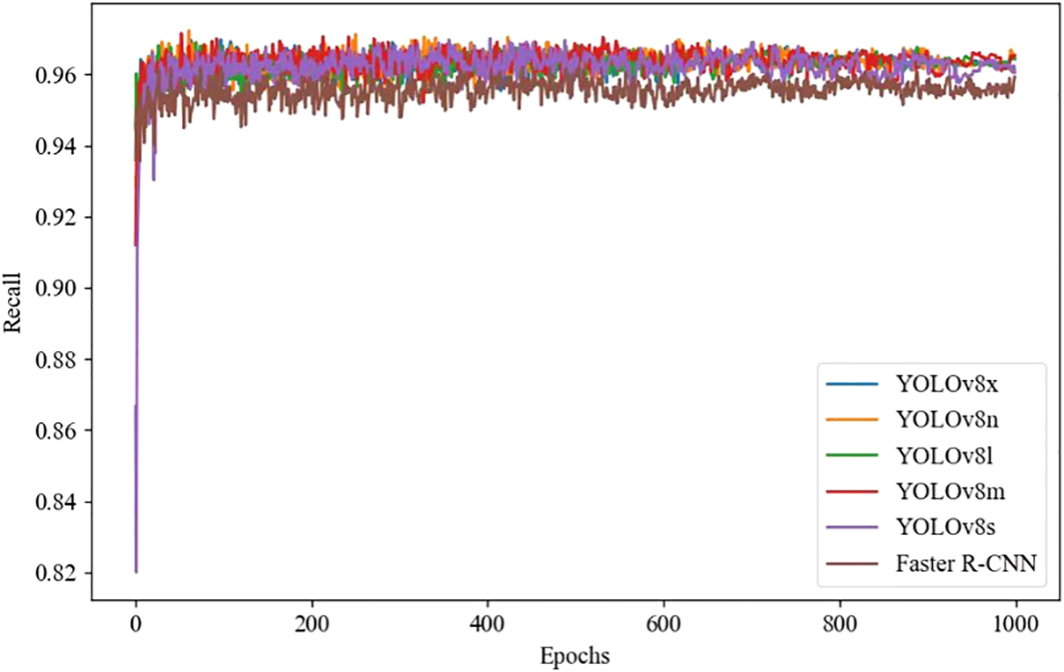
Comparison of training recall using deep learning models.

**Figure 7:**
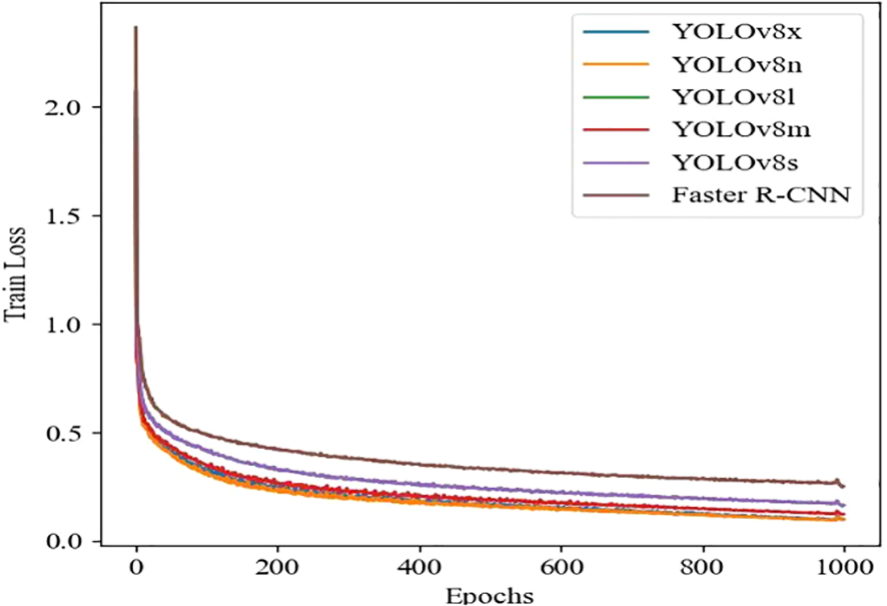
Comparison of training loss using deep learning models.

YOLOv8 and Faster R-CNN models use the following unified configuration during training: SGD optimizer is used, batch size is set to 16, input image size is 640 × 640, and training epochs are 1000. The selection of learning rate is based on the implementation of grid search method on the validation set, and the optimal initial value is determined through cross validation. Combined with the learning rate attenuation strategy, the convergence is accelerated. Therefore, the learning rate is set to 0.001. During the training process, an early stopping strategy is adopted to prevent overfitting, and the model usually converges in hundreds of rounds. The learning rate and training loss results of different deep learning models are shown in **Figures 5**, **6**, **7**, where the horizontal axis represents the number of training iterations, the vertical axis in **Figure 5** represents accuracy, the vertical axis in **Figure 6** represents recall, and the vertical axis in **Figure 7** represents training loss. **Figures 5**, **6** shows that as the number of training iterations increases, the accuracy and recall of different deep learning models gradually stabilize after 200 epochs, but the accuracy and recall of the YOLOv8n model increase and stabilize the fastest, indicating that the YOLOv8n model has better performance throughout the entire training process. **Figure 7** shows that as the number of iterations increases, the training loss of different deep learning models gradually decreases after 200 epochs, and then converges and remains stable. The YOLOv8n model achieves the best convergence performance.

### Results analysis of spike grain counting

To verify the detection performance of different models on wheat grains, this study conducted comparative experiments on YOLOv8n, YOLOv8s, YOLOv8m, YOLOv8l, YOLOv8x, and Faster R-CNN based on a self-built wheat grain image dataset, the results are shown in **Table 2**. From **Table 2**, it can be seen that YOLOv8n has higher precision, recall, mAP50, and mAP50-95, which are 96.8%, 96.8%, 98.9%, and 58.4%, respectively. Faster R-CNN has lower precision, recall, mAP50, and mAP50-95, with values of 95.7%, 95.5%, 97.7%, and 57.1%, respectively. In terms of precision, the precision of different models ranges from 95.7% to 96.8%. The precision of YOLOv8n, YOLOv8s, YOLOv8m, YOLOv8l, YOLOv8x, and Faster R-CNN is 96.8%, 96.5%, 96.7%, 96.3%, 96.2%, and 95.7%, respectively. In terms of recall, the recall rates of different models ranged from 95.5% to 96.8%. The recall of YOLOv8n, YOLOv8s, YOLOv8m, YOLOv8l, YOLOv8x, and Faster R-CNN were 96.8%, 96.1%, 96.5%, 96.3%, 96.7%, and 95.5%, respectively. In terms of mAP50, the mAP50 of different models ranges from 97.7% to 98.9%. The mAP50 of YOLOv8n, YOLOv8s, YOLOv8m, YOLOv8l, YOLOv8x, and Faster R-CNN are 98.9%, 98.4%, 98.7%, 98.8%, and 97.7%, respectively. Although YOLOv8 improves the counting accuracy of wheat grains and effectively reduces errors caused by dense overlap and adhesion; The errors generated mainly stem from the model’s incorrect recognition of glumes and ears without grains. In summary, YOLOv8n has high precision, recall, mAP50, and mAP50–95 in wheat spike grain detection, significantly improving the recognition ability of wheat spike grain.

**Table 2:** Results analysis of spike grain counting using different deep learning models.

Models	Precision %	Recall %	mAP50 %	mAP50-95 %
YOLOv8n	96.8	96.8	98.9	58.4
YOLOv8s	96.5	96.1	98.4	57.7
YOLOv8m	96.7	96.5	98.7	57.5
YOLOv8l	96.3	96.3	98.7	57.7
YOLOv8x	96.2	96.7	98.8	57.2
Faster R-CNN	95.7	95.5	97.7	57.1

### Visualization analysis of spike grain counting

**Figure 8:**
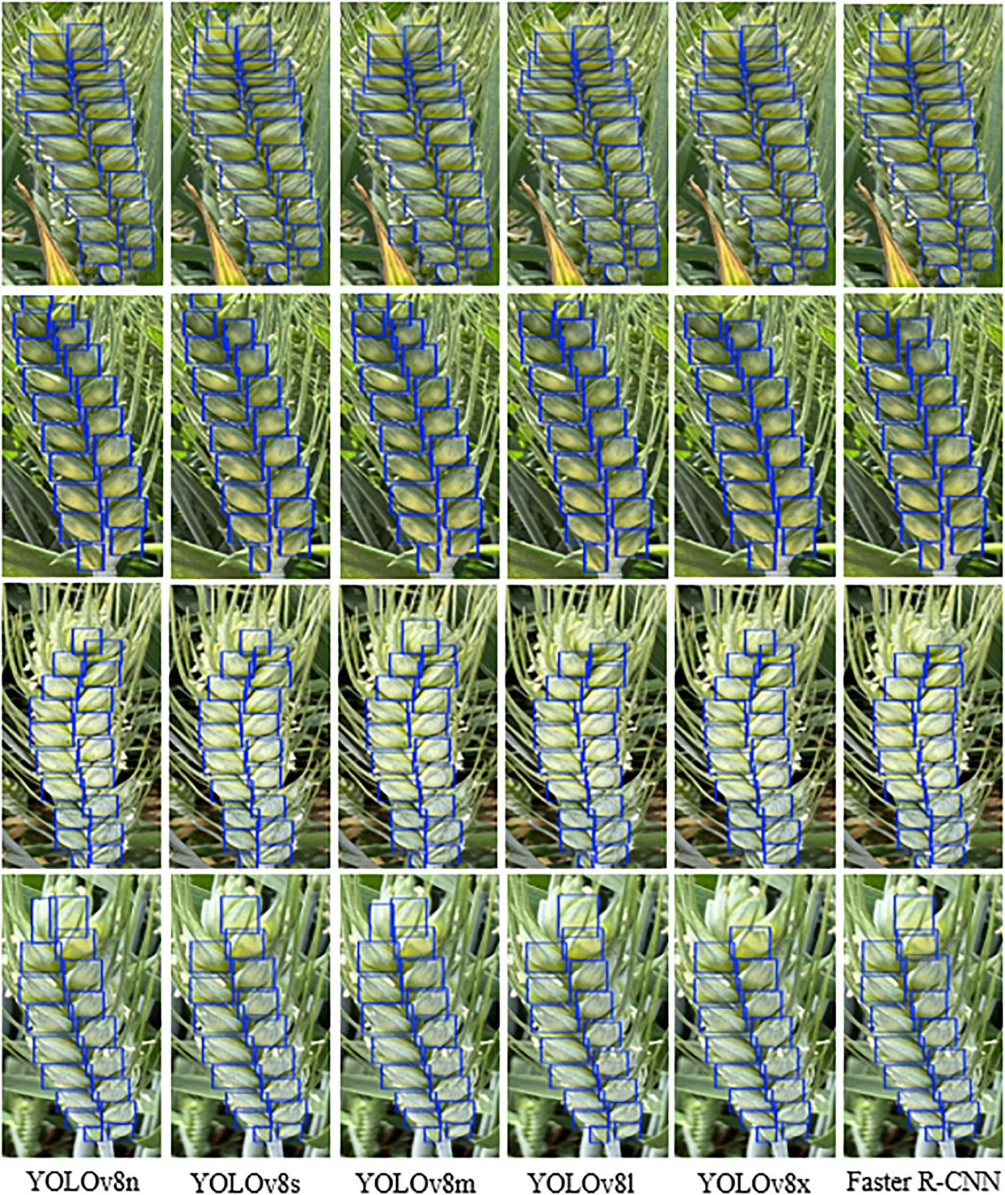
Visual analysis of spike grain counting using different deep learning models.

In order to more intuitively demonstrate the detection performance of different models on wheat spike grain images under natural field conditions, this study visualized the recognition results of wheat spike grain images, where the blue box represents the detection box predicted by the model, as shown in **Figure 8**. **Figure 8** shows the visualization results of grain counting for different models from left to right, namely YOLOv8n, YOLOv8s, YOLOv8m, YOLOv8l, YOLOv8x, and Faster R-CNN. The phenomenon of missed detections in Faster R-CNN is more obvious, and YOLOv8s has reduced the number of missed detections of wheat grains compared to YOLOv8l and YOLOv8x, especially in areas with dense grain arrangement, adhesion, and occlusion. Compared with the above five models, YOLOv8n improved the recognition ability of wheat grains. Therefore, YOLOv8n exhibits higher counting accuracy and better generalization ability for spike grain regions with small size, dense distribution, and complex background.

### Performance analysis of spike grain counting

**Table 3** shows the parameter quantities of six models FLOPs, Comparison of inference time, model size, and GPU memory usage. Among them, parameter count, FLOPs, and GPU memory usage refer to the information obtained during model training, while inference time refers to the computation time required for the trained model to process a single input image and generate prediction results. Model size refers to the space occupied by the trained model during storage. From **Table 3**, it can be seen that YOLOv8x has the highest parameter count, computational complexity, and GPU memory usage, at 68.2M, 257.8B, and 519.02MB, respectively. YOLOv8n has the lowest, at 3.2M, 8.9B, and 162.20MB, respectively. In terms of inference time and model size, Faster R-CNN has the highest, at 67.17ms and 108.6MB, respectively, while YOLOv8n has the lowest, at 14.54ms and 6.24MB, respectively. It can be seen that YOLOv8n not only has a lower parameter count FLOPs, inference time, model size, and GPU memory usage, as well as higher detection accuracy in wheat spike grain counting tasks, fully meet the grain counting requirements of wheat breeding.

**Table 3:** Performance analysis of spike grain counting using different deep learning models.

Models	Parameters /M	FLOPs /B	Inference time/ms	Model size/MB	GPU memory usage/MB
YOLOv8n	3.2	8.9	14.54	6.24	162.20
YOLOv8s	11.2	28.6	16.75	21.5	202.40
YOLOv8m	25.9	78.9	26.05	49.7	267.40
YOLOv8l	43.7	165.2	34.41	83.7	376.43
YOLOv8x	68.2	257.8	55.09	131	519.02
Faster R-CNN	27.2	470.6	67.17	108.6	460.80

### Fitting analysis of spike grain counting

Fifty different wheat variety grain images were randomly selected, and the predicted spike grain number and measured spike grain number obtained from the model were fitted and analyzed. The relationship between the predicted grain number and the measured grain number is shown in **Table 4**. The model directly outputs the bounding boxes and corresponding category probabilities for each wheat grain. It is necessary to first filter out low confidence false positive detection boxes through confidence filtering, then merge overlapping detection boxes through non maximum suppression, and finally count the remaining bounding boxes to obtain the predicted grain number. The model prediction flow first outputs all detected wheat grain boundary boxes in a single image, and then calculates the predicted total grain number of the image by counting the total number of boundary boxes. Therefore, the calculation of RMSE is based on the deviation between the predicted total and the true total for each image. From **Table 4**, it can be seen that the R^2^ values of the six models are between 0.67-0.94. YOLOv8n has the best fitting effect with an R^2^ of 0.94 and RMSE of 1.02; the fitting effect of Faster R-CNN is relatively poor, with an R^2^ of 0.67 and RMSE of 1.40. This indicates that YOLOv8n can effectively meet the practical needs of spike grain counting and localization, demonstrating strong robustness.

**Table 4:** Fitting analysis of spike grain counting using different deep learning models.

Models	Regression	R^2^	RMSE
YOLOv8n	y=1.0038x−1.105	0.94	1.02
YOLOv8s	y=0.985x−0.7589	0.82	1.17
YOLOv8m	y=0.8625x+3.6131	0.78	1.25
YOLOv8l	y=1.003x−1.3559	0.79	1.39
YOLOv8x	y=0.8462x+4.147	0.81	1.25
Faster R-CNN	y=0.831x+4.8889	0.67	1.40

### Image analysis of typical spike grain

**Figure 9:**
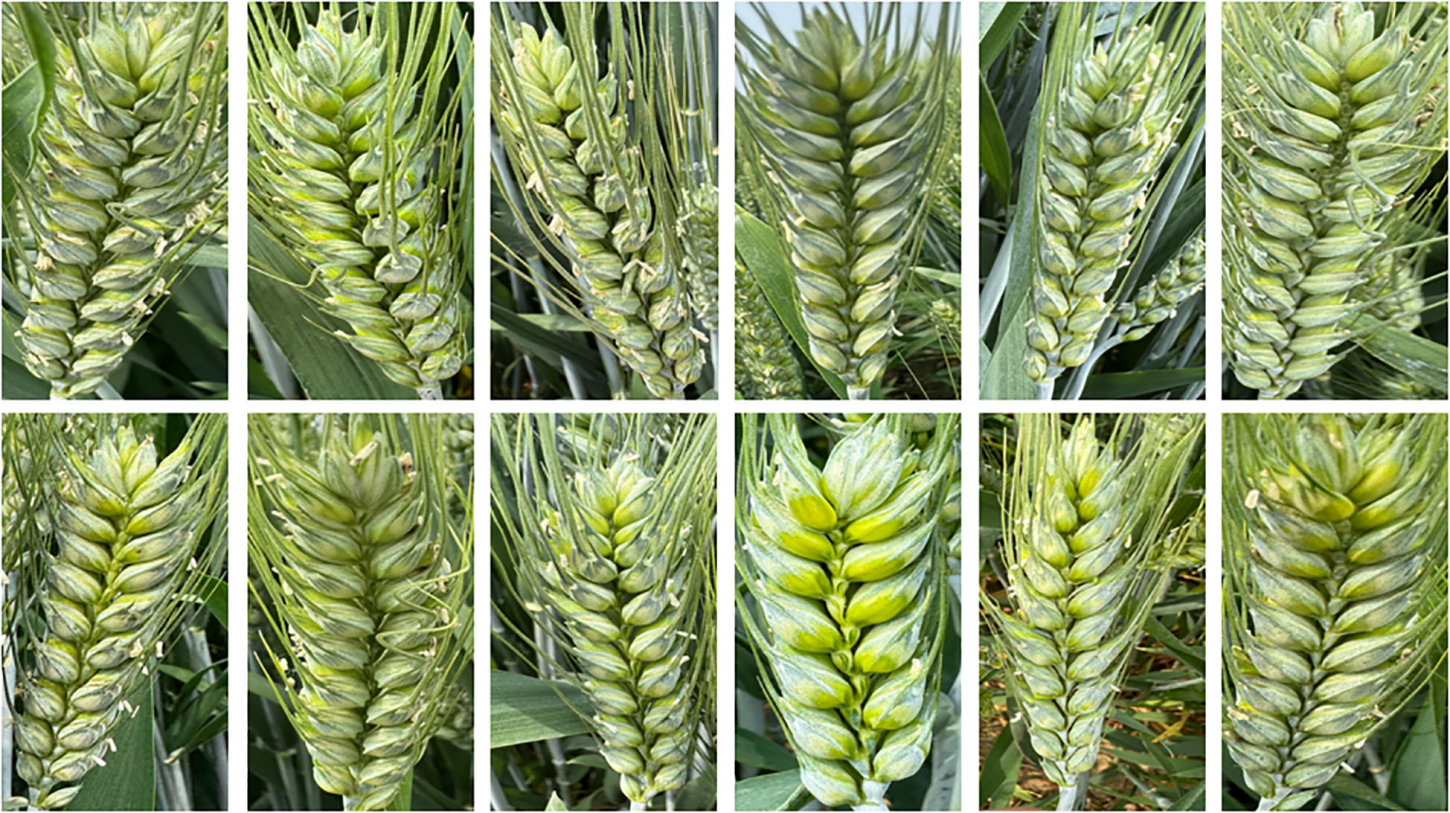
Typical image of wheat spike grain.

Affected by factors such as imaging angle and wheat spike density, **Figure 9** shows typical images in the test set. In complex field environments, wheat spike grain detection and counting face severe challenges. Under natural conditions, wheat spike grains overlap and adhere to each other, and leaves and awns block key grain positions, making target detection difficult; the obstruction of wheat grains by spikes and husks results in some targets being unable to correctly detect wheat spike grains. In addition, in complex field environments, there is severe obstruction between wheat leaves, stems, and grains, and uneven lighting can easily form reflective or backlit areas; there is a high degree of color overlap between the grains, leaves, and background, and the color similarity between small-sized grains and the background is significant, which further increases the difficulty of wheat spike grain detection and restricts the improvement of wheat spike grain counting accuracy.

### Five fold cross validation and analysis of variance

In order to prevent overfitting of the model and comprehensively evaluate its generalization ability, five fold cross validation was chosen to train and validate different subsets of data. Five fold cross validation ensures that the proportion of samples in each compromise category is exactly the same as that of the original dataset during partitioning. The selfbuilt wheat seedling dataset is randomly divided into five subsets of similar size, with four subsets selected as the training set and the remaining one as the validation set. Random seeds are fixed, and their core role in cross validation is to ensure that the data partitioning results are reproducible, using the same augmentation between folds for training.

The five fold cross validation results are shown in **Table 5**. The Precision of YOLOv8n model is 96.4-96.8%, Recall is 96.0-96.8%, mAP50 is 97.6-98.9%, and mAP50–95 is 57.0-58.4%. The analysis of variance results showed that the F-values of Precision, Recall, and mAP50–95 for different deep learning models were 23.32, 17.75, and 4.27, respectively, reaching a highly significant level.

**Table 5:** Five-fold cross validation results of different deep learning models.

Models	Five-fold validation	Precision /%	Recall /%	mAP50 /%	mAP50-95 /%
YOLOv8n	One fold	96.8	96.8	98.9	58.4
	Two fold	96.7	96.3	97.7	57.9
	Three fold	96.4	96.2	97.6	57.8
	Four fold	96.5	96.2	97.7	57.0
	Five fold	96.5	96.0	97.6	57.1
YOLOv8s	One fold	96.5	96.1	98.4	57.7
	Two fold	96.5	96.5	97.9	56.8
	Three fold	96.7	96.2	97.8	56.8
	Four fold	96.6	96.3	97.8	56.9
	Five fold	96.2	96.6	97.9	56.9
YOLOv8m	One fold	96.7	96.5	98.7	57.5
	Two fold	96.1	96.5	97.8	56.6
	Three fold	96.4	96.2	97.7	56.6
	Four fold	96.2	96.3	97.7	56.5
	Five fold	96.3	96.4	97.7	56.4
YOLOv8l	One fold	96.3	96.3	98.7	57.7
	Two fold	96.1	96.4	97.5	56.5
	Three fold	96.3	96.5	97.8	55.3
	Four fold	95.9	96.2	97.5	54.5
	Five fold	96.4	96.2	97.5	55.3
YOLOv8x	One fold	96.2	96.7	98.8	57.2
	Two fold	96.1	96.4	97.6	56.1
	Three fold	96.3	96.2	97.6	56.5
	Four fold	96.2	96.2	97.7	56.4
	Five fold	96.4	95.9	97.4	55.8
Faster R-CNN	One fold	95.7	95.5	97.7	57.1
	Two fold	95.4	95.1	97.5	56.8
	Three fold	95.2	95.2	97.5	56.9
	Four fold	95.4	95.5	97.4	56.8
	Five fold	95.6	95.3	97.8	57.2
	F value	23.32**	17.75**	0.48	4.27**

**indicates significant differences at the 0.01 level.

### Verification of test results

To evaluate the performance of YOLOv8n, YOLOv8s, YOLOv8m, YOLOv8l, YOLOv8x, and Faster R-CNN models in the automatic counting of wheat grains, 20 wheat grain images were randomly selected from the test set, and the accuracy was compared between manually annotated true values and predicted values of each model. From **Table 6**, it can be seen that the average relative error of YOLOv8n counting is 3.6%. Compared with YOLOv8s, YOLOv8m, YOLOv8l, YOLOv8x, and Faster R-CNN, YOLOv8n has decreased by 0.4%, 0.2%, 1.0%, 0.5%, and 2.6%, respectively, indicating that the overall prediction counting error of YOLOv8n is smaller and the grain counting results are more stable.

**Table 6:** Verification of spike counting results of different deep learning models.

Image number	True value	YOLOv8n	Relative error/ %	YOLOv8s	Relative error/ %	YOLOv8m	Relative error/ %	YOLOv8l	Relative error/ %	YOLOv8x	Relative error/ %	Faster R-CNN	Relative error/ %
1	40	40	0	38	5.0	38	5.0	40	0	42	5.0	36	10.0
2	36	34	5.6	34	5.6	38	5.6	34	5.6	36	0	38	5.6
3	40	40	0	42	5.0	40	0	38	5.0	40	0	38	5.0
4	36	38	5.6	36	0	36	0	36	0	34	5.6	34	5.6
5	40	38	5.0	40	0	42	5.0	40	0	38	5.0	42	5.0
6	34	34	0	34	0	34	0	36	5.9	32	5.9	32	5.9
7	36	34	5.6	32	11.1	34	5.6	38	5.6	36	0	36	0
8	38	38	0	38	0	38	0	36	5.3	40	5.3	34	10.5
9	40	38	5.0	36	10.0	38	5.0	42	5.0	38	5.0	40	0
10	44	42	4.5	44	0	44	0	40	9.1	46	4.5	42	4.5
11	44	44	0	46	4.5	41	6.8	44	0	42	4.5	46	4.5
12	38	40	5.3	38	0	40	5.3	38	0	36	5.3	36	5.3
13	40	42	5.0	38	5.0	40	0	38	5.0	40	0	38	5.0
14	36	34	5.6	36	0	36	0	34	5.6	38	5.6	32	11.1
15	30	30	0	30	0	30	0	28	6.7	32	6.7	28	6.7
16	40	38	5.0	42	5.0	36	10.0	40	0	40	0	42	5.0
17	40	38	5.0	38	5.0	42	5.0	38	5.0	42	5.0	36	10.0
18	42	42	0	44	4.8	44	4.8	40	4.8	42	0	40	4.8
19	40	40	0	40	0	40	0	42	5.0	38	5.0	38	5.0
20	40	40	0	42	5.0	38	5.0	38	5.0	40	0	40	0
Average			2.9		3.3		3.1		3.9		3.4		5.5

## Spike grain counting system

### Overall architecture

**Figure 10:**
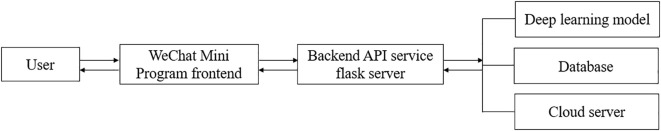
Overall structure of wheat grain counting system.

In order to enable agricultural researchers to quickly and conveniently count the number of wheat spike grain in practical work, this study deployed an wheat grain counting model to mobile devices, thereby achieving automatic calculation of the number of wheat spike grain. This study is developed based on the WeChat mini program platform, using a microservice architecture consisting of a WeChat mini program front-end, back-end server, and deep learning model. The front-end of WeChat Mini Program obtains images through Mini Program API and sends them to the back-end service of WeChat Mini Program through HTTP request. The backend of the WeChat mini program requests verification and forwards the image to the server for processing. The server-side uses Python language to preprocess the received images and input them into the wheat grain counting model deployed on the server. Compared to traditional applications, WeChat mini programs have lower development costs and are cross platform, allowing them to run on multiple operating systems such as iOS and Android. WeChat mini programs access backend service interfaces through domain names, achieving data transmission and effectively ensuring system stability and scalability. The database uses WeChat mini program cloud database, which supports real-time synchronization of data changes to the front-end, significantly improving the user experience; Simultaneously providing high-performance data storage and retrieval capabilities, ensuring that the mini program responds quickly to user requests. WeChat cloud database is built on the tencent cloud platform, relying on a powerful underlying architecture to provide high-performance storage and retrieval capabilities for mini programs. Through advanced storage algorithms and index optimization techniques, cloud databases can quickly respond to various data requests from users in mini programs, whether it is complex query operations or frequent data read and write, ensuring smooth operation of mini programs. Combining WeChat cloud development and tencent cloud platform, we provide convenient and efficient solutions for mini programs in terms of data management and computing resources. The overall structure of the wheat grain counting system is shown in **Figure 10**.

### Module function

The WeChat mini program mainly includes modules for uploading images, displaying results, and recording history. Users can upload wheat spike grain images from photo or local photo albums to the WeChat mini program page, call the wheat grain counting model, preprocess and count the images, display the predicted values and visualized wheat spike grain images, and save the images to the WeChat mini program cloud database. The history module allows users to view previous image records anytime and anywhere, including image counting time, original images, visualized images, and predicted quantities, greatly improving work efficiency. The calculation time for a single wheat grain image on the mobile end is about 0.5–1 s, and the input image size is uniformly 640×640 pixels. The WeChat mini program uploads images to the server through an API, and the server uses a task queue mechanism to schedule and manage recognition requests. During the upload and recognition process, the front-end displays the processing stage in real time through animation effects, and automatically triggers a retry mechanism in case of network abnormalities. The deep learning model outputs visual images and prediction results, and returns them to the WeChat mini program front-end. After receiving the images and prediction results, the front-end displays them on the front-end page and saves the images to the WeChat mini program cloud database for users to view.

**Figure 11:**
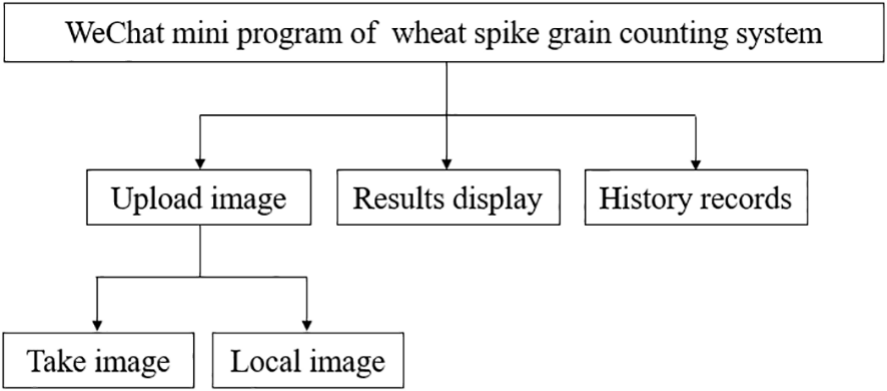
Functional design of spike grain counting system.

The wheat grain counting WeChat mini program designed in this study, After long-term testing and continuous updates, the wheat spike grain counting system has been designed reasonably, with a simple interface operation and easy use, meeting the actual needs of wheat spike grain counting and improving the accuracy and robustness of wheat spike grain detection. The functional design of the wheat spike grain counting system is shown in **Figure 11**.

### Database and backend server design

Installing Anaconda can avoid dependency conflicts between different software packages and make Python environment management more flexible. The model code was developed in PyCharm and registered as a domain name on the Tencent Cloud official website for filing. At the same time, apply for an SSL certificate and configure the HTTPS protocol to encrypt network communication and prevent data from being eavesdropped or tampered with during transmission. The backend server is built on the Flask framework and is a lightweight web application framework that supports flexible routing definitions. It can receive and process HTTPS requests from WeChat mini programs, perform operations including database queries and business logic processing. Ultimately, deploying backend services under a specific domain ensures the security and reliability of communication between WeChat mini programs and servers.

By creating a Flask application instance, the model initialization is completed and loaded onto the GPU to accelerate the computation process. The backend server receives the image URL in the request, preprocesses the image, and passes the model inference. After processing, the system encapsulates the visualization results and predicted data in dictionary form and returns them to the front-end in JSON format, thereby achieving efficient data transmission and display. After parsing the JSON response on the frontend mini program page, it dynamically displays the visualization effect of user uploaded images and the predicted quantity of the model, providing users with intuitive results presentation. At the same time, the system will link and store the generated visual images in a cloud database, making it easier for researchers to trace historical records and achieve persistent data management. The overall software architecture emphasizes the simplicity of user operations and the intuitiveness of the interface.

In terms of data management, Tencent Cloud provides a convenient database operation interface for mini programs, supporting developers to easily add, delete, modify, and query data, and to achieve real-time management and maintenance of data through a visual console. In terms of computing resources, relying on Tencent Cloud’s powerful elastic scalability, the system can dynamically allocate resources according to the actual business load of mini programs, effectively ensuring service stability and response efficiency in high concurrency scenarios.

In the image upload process, to optimize transmission efficiency, the system first uses JPEG or PNG compression algorithms to preprocess the original image, effectively reducing the image size while ensuring image quality, and uniformly converting the compressed image to WebP format. This format significantly reduces the file size compared to traditional JPEG or PNG formats under the same visual quality, thereby greatly shortening the image transmission time and improving the smoothness of image uploading for users.

At the data transmission level, the system uses SSL encryption technology to encrypt all transmitted data, converting plaintext data into ciphertext data, effectively preventing man in the middle attacks and data leaks, and ensuring the confidentiality and integrity of information in network transmission. At the same time, the platform implements strict user authentication and authorization mechanisms to ensure data security and user privacy from the source of access.

At the server-side processing level, introducing message queues for asynchronous management of detection requests improves the stability of the system in high concurrency scenarios. To further enhance robustness, the system integrates an automatic retry mechanism: if image processing is interrupted due to temporary failures (such as network jitter or instantaneous service unavailability), it will automatically attempt to re execute the task, effectively preventing task loss. For cases where automatic recovery is still not possible after a retry, the system promptly provides feedback to users, facilitating troubleshooting and improving the maintainability and user experience of the system.

In terms of data storage and backup, the system relies on cloud databases to permanently store images and related business data. By setting a regular automatic backup mechanism, the risk of data loss caused by unexpected situations can be effectively prevented. This set of data management strategies not only ensures data consistency, security, and long-term reliability, but also provides stable underlying support for the system’s efficient image processing capabilities.

## System implementation

### Register and login

First use requires registering an account. After clicking on registration, please enter your phone number and set a password. The relevant information will be securely saved to the WeChat mini program cloud database. After successful login, the personal page will display the bound phone number and provide an option to log out. When the mini program is opened again, the system will automatically read the locally cached phone number information and complete the quick login. If you click exit, the page will return to the login interface, where you can enter your registered phone number and password to log in again. The function page provides three main operation entrances, namely: uploading images, displaying results, and the history module, making it convenient for users to quickly use the corresponding functions.

### Spike grain counting

After clicking the grain count button, the system will request camera permission. The user clicks agree to start the camera. Aim your phone at the grain to be tested when taking photos. After the photo shoot is completed, the frontend of the mini program will send it to the server via HTTPS request. After receiving the image, the server performs image preprocessing and deep learning model analysis in sequence. After processing, the system will store the user’s phone number, original image, visualization results, and recognized grain number in the cloud database. At the same time, the results will be packaged in JSON format and returned to the frontend of the mini program through HTTPS response.

If the desired image has been saved in the phone’s photo album, you can click the local album button. The system will pop up a prompt for album permission application. Click agree to jump to the album page. Select the grain image to be recognized and upload it. After waiting for a few seconds, the page will display the original image, visual analysis results, and the number of grains recognized simultaneously.

Click the history button on the homepage, and the system will query the associated image records in the cloud database based on the currently logged in phone number. After the query is completed, the relevant records will be displayed on the page in the form of a list. Click on any record to jump to the counting details page of that image. The details page includes recognition time, original images, visual analysis results, and the predicted number of grains per ear by the model.

## Discussion

As the latest version of the YOLO series, YOLOv8 released in 2023 and has achieved a breakthrough in speed and precision in object detection tasks. In contrast, Faster R-CNN is as a classic two-stage detection algorithm, significantly improves detection precision by introducing region proposal network. In the process of wheat spike grain image acquisition, factors such as camera equipment, shooting angle, and weather conditions can all affect the imaging quality. The differences in image quality obtained by different shooting devices will directly affect the subsequent image processing results. Taking wheat grain images in strong or weak light environments can result in abnormal exposure or shadow interference, which can affect later data processing. In addition, adverse weather conditions not only increase the difficulty of data collection, but may also lead to missing the optimal collection time during critical reproductive periods. This study innovatively integrates the collaborative advantages of automated image processing and deep learning, automatically optimizing image processing parameters through deep learning models, significantly reducing manual intervention in the feature extraction process, and improving image analysis efficiency. Automated image processing can provide a large amount of annotated datasets for deep learning models, reducing the workload of data annotation, improving annotation accuracy, and thus enhancing model training efficiency. On the premise of ensuring the accuracy of data collection and analysis, the problems of outdated traditional wheat breeding spike grain data collection methods, time-consuming and laborious data recording, and complex data management have been solved, significantly reducing the field labor intensity of breeding researchers and improving the efficiency of wheat grain counting.

In field experiment, wheat phenotypic traits are mainly influenced by genotype and growth environment, which is an important bridge for studying the mechanism of “genotype-phenotype-environment”. Currently, research on wheat grain counting mostly uses manual counting methods after threshing, which have low efficiency, large workload, high cost, and cannot achieve non-destructive measurement. Research has shown that based on micro CT scanning to reconstruct the three-dimensional structure of 18 wheat spikes, there is a high correlation between the number of wheat grains detected and the actual number of grains, with R² of 0.989. However, this method is expensive, has a complex and time-consuming operation process, and has obvious limitations (Zhou et al., 2021). Therefore, this study relies on regional trials of wheat varieties and uses smartphones to collect images, which significantly improves the convenience and practicality of data collection, different wheat varieties have varying characteristics such as grain size, sparsity, plumpness, spike length, and wheat awn. Generally, wheat with larger spike types, sparse arrangement, and no awns has relatively lower difficulty in identifying wheat spike grain. However, wheat with smaller spike types, dense arrangement, and awns has higher difficulty in identifying wheat spike grain. In the case of extremely dense samples, the probability of wheat spike overlap is high, and the regression idea of YOLO algorithm is based on dividing the image into grids, that is, each grid can only predict at most one target object. Therefore, it performs poorly in the case of multiple target objects appearing in the same grid, and cannot recognize all multiple targets. In wheat spike detection, some images have overlapping wheat spikes that are not recognized, and adjacent wheat spikes are not recognized. Two wheat spikes closely connected are recognized as one wheat spike. This study not only directly obtained the number of wheat spike grain, but also eliminated indirect measurement errors; Meanwhile, deep learning methods can efficiently identify a large number of samples with high accuracy. The results showed that this method can achieve faster and more accurate wheat spike grain counting.

In the process of wheat breeding, automatic counting of spikes and grains is currently the bottleneck of research, and its low counting accuracy is a key factor restricting the progress of breeding. The grain filling period is as a critical period determining the of wheat grain weight, and is crucial for yield formation. Xu et al. (2023) proposed a wheat grain counting model CBAM-HRNet based on CBAM, with an R^2^ of 0.85 and an average relative error of 2.09%. Alkhudaydi et al. (2019) used the fully convolutional neural network Spikelet FCN to extract wheat grains, and its counting error was reduced by 89% compared to traditional methods. Li and Wu (2022) proposed an improved YOLOv5 algorithm based on shallow feature layers, with an mAP of 94.3%, accuracy of 88.5%, and recall of 98.1%. Zang et al. (2022) proposed an improved YOLOv5s wheat spike detection method based on attention mechanism, which can accurately detect small-scale wheat spikes and effectively solve the problems of wheat spike occlusion and cross overlap. Wu et al. (2020) used transfer learning methods to optimize the detection and counting model of wheat grains, with an average accuracy of 0.91. Geng et al. (2025) proposed the wheat spike grain point-to-point network WSG-P2PNet, with MAE of 1.72 and an RMSE of 2.35. Geng et al. (2024) proposed the wheat grain counting model SGCountM, with an RMSE of 4.38 and an R^2^ of 0.85. In the past, these studies mainly focused on wheat grain number detection under different cultivation and management measures, and the data was usually not based on wheat grain images collected *in situ* in the field, and there was a common problem of limited sample size. This study innovatively integrates the collaborative advantages of automated image processing and deep learning to address key challenges such as small grain size, dense distribution, diverse morphology, and susceptibility to occlusion in wheat breeding scenarios. This effectively improves the detection ability of difficult samples, significantly reduces the missed detection rate of dense targets, and enhances the generalization performance of the model in different environments. This study selected six state-of-the-art deep learning models that can accurately and effectively detect the number of wheat spike grain during the grain filling stage, which can be used to replace traditional manual counting methods. In all wheat spike grain tests, YOLOv8n showed high precision, recall, and mAP50, with values of 96.8%, 96.8%, and 98.9%, respectively. At the same time, YOLOv8n solves the problem of false counting and omission caused by occlusion, overlap in spike grain image detection, thus efficiently and accurately counting the number of wheat spike grain, which can provide technical reference for spike grain counting and yield estimation in actual production.

The wheat grain counting model can directly obtain the number of grains per ear, which reduces indirect errors to a certain extent. However, there are still some errors in the model, mainly due to the misjudgment of wheat husks and the presence of spikes but no grains by deep learning models. This problem needs to be further solved through innovative methods. Wheat growth is a complex and dynamic process, manifested in the periodic changes in grain color characteristics and the diversity of field backgrounds. Most existing object detection models are usually optimized at specific growth stages, making it difficult to generalize to other stages or different field environments, especially for detecting small targets, high-density, and multi shaped objects. As shown in **Figure 9**, the wheat grain image itself has high complexity, with severe occlusion, adhesion, and overlap between grains, making it difficult for traditional methods to effectively extract features and distinguish grains from the background. The above factors collectively increase the difficulty of wheat grain number detection, making conventional detection models prone to missed and false detections when applied to wheat grain images. In contrast, the YOLOv8n model used in this study exhibits better robustness and detection stability in such complex scenarios.

This study used an object detection model to achieve rapid detection and counting of wheat spike grain during the grain filling stage in a field environment. However, the robustness of methods based on manually designed features is still insufficient when dealing with scenarios of wheat spike grain adhesion and growth variation. Especially for small-sized grains with high-density distribution, they often suffer from serious occlusion, overlap, and cross growth, leading to frequent false and missed detections in wheat spike grain detection. At present, there are relatively abundant publicly available wheat spike grain dataset, but specialized datasets for wheat spike grain is relatively scarce. Compared to counting wheat spike, the task of counting spike grain is more challenging, because wheat spike grain is smaller, more densely distributed, and have strong adhesion between grains. Existing research methods are mostly based on images of threshed wheat spike grain collected in laboratory environments without complex backgrounds. Therefore, this study constructed a wheat spike grain dataset with complex field scenes. In the future, we need to collect wheat spike grain image across regions, varieties, and growth stages, covering different shooting angles, resolutions, and equipment types, to construct a large-scale dynamic dataset of wheat grain. Based on different datasets and application scenarios, combined with multiple object detection models, further optimize the models and algorithms to cope with complex field scenarios and achieve higher counting accuracy and robustness of wheat spike grain. Combining the wheat grain counting method with drone technology to achieve accurate detection of wheat spike grain on a large scale. At the same time, we will focus on the field of wheat spike grain detection, conduct research on the migration and generalization of intelligent wheat spike grain detection algorithms, and develop lightweight wheat spike grain intelligent detection software.

## Conclusion

This study successfully evaluated six state-of-the-art deep learning models for wheat spike grain detection and counting, all of which were able to accurately count wheat spike grain. The YOLOv8n is the best performing model, with the highest predictions for precision, recall and mAP50, at 96.8%, 96.8% and 98.9%, respectively. The performance of other models is also satisfactory, among which YOLOv8s, YOLOv8l, YOLOv8m, YOLOv8x, and Faster R-CNN have prediction precision between 95.7-96.7%, and mAP50 is between 97.7-98.7%. YOLOv8n not only has a lower number of parameters FLOPs, inference time, model size, and GPU memory usage, as well as higher detection precision in wheat spike grain counting tasks, fully meet the spike grain counting requirements of wheat breeding. This study designed and implemented a WeChat mini program for wheat spike grain counting, which achieved automatic detection and counting of wheat spike grain, providing valuable reference for spike grain detection, counting and yield estimation of other crops.

This study can provide a new technological means for wheat breeding, which has good practical value and application prospects in improving the efficiency and accuracy of wheat spike grain number identification. In response to the problems of false detection and missed detection of wheat spike grain caused by high density, severe occlusion, and cross overlap, this study obtained a training model through object detection algorithm and achieved automatic detection and counting of wheat spike grain. The aim is to provide technical support for the statistical analysis of spike grain number and yield estimation of new wheat varieties in the field. Meanwhile, this method also provides valuable reference for grain detection, counting, and yield estimation of other crops. In future research, we should integrate multimodal data, construct and open up a shared wheat spike grain image dataset, and develop an image-based comprehensive estimation method for wheat yield. This study not only significantly reduces the complexity, subjectivity, and time cost of manual field investigations, but also improves the efficiency of wheat spike grain counting. It will provide scientific value and practical support for intelligent breeding, yield intelligent evaluation, and high-throughput phenotype automation analysis.

## Data Availability

The data analyzed in this study is subject to the following licenses/restrictions: The raw data supporting the conclusions of this article will be made available by the authors, it will be released upon acceptance. Requests to access these datasets should be directed to the correspondence.
